# Recurrent Subdural Hematoma: An Institutional Experience

**DOI:** 10.7759/cureus.42582

**Published:** 2023-07-27

**Authors:** Sai Sriram Swamiyappan, Visvanathan Krishnaswamy, Vivek Visweswaran, Sangeetha A, Rav Tej Bathala, Harsh Karnati, Jayesh Gupta, Ganesh K

**Affiliations:** 1 Neurosurgery, Sri Ramachandra Institute of Higher Education and Research, Chennai, IND; 2 Neurological Surgery, Sri Ramachandra Institute of Higher Education and Research, Chennai, IND

**Keywords:** subdural hematoma, ct prediction, burr hole drainage, open craniotomy, recurrence rate

## Abstract

Background

Chronic subdural hematoma (CSDH) is a common neurosurgical problem, which offers a good outcome following surgery. In many cases, burr hole irrigation and drainage under local anesthesia can provide satisfactory results. However, recurrence can be a cause for concern for both the surgeon and the patient. While recurrence is not a frequent phenomenon, studies have reported rates of up to 31.6%.

Aims and objectives

In this study, our objective is to examine a comprehensive range of potential risk factors and provide valuable insights into identifying patients at a higher risk of recurrence to aid in surgical decision-making.

Methodology

This study employed a prospective and retrospective design, conducted between 2017 and 2021, at Sri Ramachandra Institute of Higher Education and Research. The study received ethical approval from the Institutional Ethics Committee. The research aimed to assess patients who underwent surgery for CSDH, with a particular focus on those who experienced recurrence.

Results

The average age of patients with recurrence was 71.5 years compared to 65.2 years in the no-recurrence group, but this difference did not show a significant statistical correlation. A significant male predominance was observed, with 27 men and four women affected (out of a total of 147 men and 73 women in the study), resulting in a statistically significant p-value of 0.01. On multivariate analysis, heterogenous subtypes were a significant predictor of recurrence (OR: 8.88, 95% CI: 6.96-16.54, p = 0.01). The mean midline shift in those with recurrence was 11.4 mm compared to 7.09 mm in those without recurrence. This was a statistically significant correlation with a p-value of 0.02. Regarding those with recurrence, 24 patients underwent evacuation using two burr holes, with one placed in the frontal region and another in the parietal region. All of these patients had a subdural drain placed, which was removed on postoperative day 2. The remaining eight patients underwent a mini-craniotomy for evacuation. We had four cases of refractory CSDH, all of whom underwent the second evacuation using burr holes. Three of them underwent evacuation via craniotomy, while the family of the fourth patient did not give consent for the procedure.

Conclusion

Patient-related factors such as gender, bilateral presentation, and the presence of hypertension and radiological factors such as the presence of heterogenous subtype and a significant midline shift are clues toward a higher chance of recurrence.

## Introduction

Chronic subdural hematomas (CSDH) are a common neurosurgical problem, typically yielding favorable outcomes following surgery [1,2]. In many cases, a less invasive procedure, such as burr hole irrigation and drainage under local anesthesia, can provide satisfactory results [3,4]. However, a subset of patients experiences recurrence, which can be a cause of concern for both the surgeon and the patient. While recurrence is not a frequent phenomenon, studies have reported rates of up to 31.6% [5]. Identifying patients at risk for recurrence is crucial to guide treatment decisions and prevent its reoccurrence through more extensive procedures.

Numerous studies have attempted to identify risk factors associated with recurrence, but conflicting data have emerged [6,7]. Factors related to the patients themselves, including age, sex, comorbidities, alcohol abuse, presence of atrophic brain or bleeding tendency, and the use of antiplatelet drugs or anticoagulants, as well as factors related to the initial bleeding, such as size, radiological characteristics, and the presence of membranes, have been investigated with inconsistent findings. The choice of surgical approach, whether a single burr hole or multiple burr holes, or even a craniotomy, has also been proposed as a potential predictor of recurrence [8-10].

In this study, our objective is to examine the factors that can predict the likelihood of recurrence in CSDH patients. By investigating a comprehensive range of potential risk factors, we aim to provide valuable insights into identifying patients at a higher risk of recurrence and inform surgical decision-making processes.

## Materials and methods

Study design

This study employed a prospective and retrospective design, which was conducted between 2017 and 2021 at Sri Ramachandra Institute of Higher Education and Research. The study received ethical approval from the Institutional Ethics Committee of Sri Ramachandra Institute of Higher Education and Research with reference number CSP-MED/18/SEP46/145. The research aimed to assess patients who underwent surgery for CSDH, with a particular focus on those who experienced recurrence. Patient demographics and the classification of CSDH types, based on the Nomura et al. classification system (hypodense, isodense, layering, and mixed types), were evaluated [11].

Inclusion criteria

Patients who had previously undergone surgery for CSDH and presented with recurrence; patients with recurrence, which is defined as the presence of an ipsilateral CSDH detected on follow-up imaging, resulting in neurological deficits within three months of the previous surgery; and patients with a second recurrence who were deemed to have refractory CSDH were all included in this study.

Exclusion criteria

Patients who did not consent to participate in the study and patients with a history of prior intracranial procedures apart from ventricular shunts were excluded from the study.

Surgical procedure

Burr hole evacuation was performed either by using the previous burr hole or by creating an additional burr hole if a single burr hole was placed during the initial surgery. Following the opening of the outer membrane, a blunt-tipped infant feeding tube was inserted for saline irrigation until the effluent became clear. A closed drainage system was placed in all recurrence cases and was removed on postoperative day 2.

In cases where a hyperdense component or extensive membranes were present, a craniotomy was performed. The outer membrane was excised, while the inner membrane was often left intact to minimize the risk of cortical injury. When an adherent inner membrane prevented re-expansion of the parenchyma, it was gently fenestrated. The bone was replaced and secured with plates and screws. Subdural drains were not used in these cases.

Postoperative care and follow-up

Patients were encouraged to mobilize and were managed by keeping their heads flat and providing supplemental nasal oxygen. Typically, patients were discharged within a week. For those who were on antiplatelet drugs or anticoagulants, which were temporarily discontinued one week before surgery, the medications were resumed from postoperative day 7. Patients were routinely followed up in the outpatient department.

Statistical analysis

All statistical analyses were performed using the Statistical Package for Social Sciences version 25 (IBM Corp., Armonk, NY). Multiple logistic regression analysis was employed to predict the independent variables associated with CSDH recurrence. A Hosmer-Lemeshow test was conducted to evaluate the goodness of fit of the multivariable model. Covariates were retained in the model if their p-value was less than 0.2, and odds ratios (OR) along with their corresponding 95% confidence intervals (CI) were calculated. A two-sided p-value of less than 0.05 was considered statistically significant. A trend was defined as a p-value less than 0.2.

## Results

A total of 229 CSDH were evacuated in 220 patients during the study period. Among them, 32 recurrences were noted in 31 patients placing the recurrence rate at 13.97%. The results of our analysis are presented in Table 1.

**Table 1:** Variables analyzed with respect to recurrence GCS: Glasgow coma scale.

Characteristics	n = 24	Univariate analysis, odds ratio	95% CI	P-value	Multivariate analysis, odds ratio	95% CI	P-value
Age (years), mean	71.5 ± 12.48	1.02	0.52-1.96	0.743			
Male:Female	27m:4f	3.88	2.96-6.2	0.01	4.57	3.81-7.4	0.01
Hypertension	11	3.72	2.78-5.13	0.019	4.12	3.57-7.96	0.015
Diabetes mellitus	16	1.21	0.98-1.61	0.598			
Anticoagulation	1	0.4	0.25-0.95	0.612			
Antiplatelet drugs	2	1.41	0.73-1	0.228			
Headache	19	0.74	0.53-2.04	0.735			
Neurological deficit	9	1.73	0.91-3.89	0.4			
Seizure	7	1.88	0.77-1.09	0.2			
GCS drop	12	1.92	0.88-2.14	0.2			
Bilateral	3	2	1.5-3.66	0.4	2.46	1.98-5.47	0.03
Time since first surgery (in days)	12.12	1.34	0.87-3.46	0.2			
Midline shift (mean/per mm)	11.4	1.19	0.95-1.35	0.02	1.32	0.99-1.51	0.02
Hematoma character		8.13	5.22-11.98	0.001	8.88	6.96-16.54	0.001
Homogenous	3						
Heterogenous	29						

Patient-specific risk factors

The average age of patients with recurrence was 71.5 years compared to 65.2 years in the no-recurrence group, but this difference did not show a significant statistical correlation. A significant male predominance was observed, with 27 men and four women affected (out of a total of 147 men and 73 women in the study), resulting in a statistically significant p-value of 0.01. Among the patients with recurrence, three were using antiplatelet medications, and one was on vitamin K antagonists. However, no statistical correlation was found between the use of these medications and recurrence. Sixteen patients had diabetes mellitus, 11 had systemic hypertension, and nine patients had both conditions. Four patients had a prior cardiac history, and one had a prior stroke on the ipsilateral side. Additionally, one patient had undergone a previous ventriculoperitoneal shunt procedure. On analysis, there was an increasing trend toward recurrence in patients with hypertension (OR: 3.72; 95% CI: 2.78-5.13; p = 0.019).

There was no correlation between recurrence and the presenting complaint or the Glasgow coma scale on admission. Furthermore, of the 32 patients with recurrences, 12 presented with a drop in GCS, nine patients had motor weakness, and seven patients had new onset seizures. Nineteen patients presented with headaches. There were nine cases of bilateral CSDH of which three recurred, two on one side and one bilaterally, presenting a significant statistical trend (p = 0.04).

CSDH characteristics

Radiologically, the mean thickness of the CSDH in our study was 17.96 mm in those without recurrence compared to 19.84 mm in those with recurrence. There was no statistically significant correlation between the thickness of the CSDH and recurrence. The mean midline shift in those with recurrence was 11.4 mm compared to 7.09 mm in those without recurrence. This was a statistically significant correlation with a p-value of 0.02. After multivariate analysis, per millimeter midline shift remained a significant predictor of recurrence (OR: 1.32, 95% CI: 0.99-1.5, p = 0.02).

**Figure 1:**
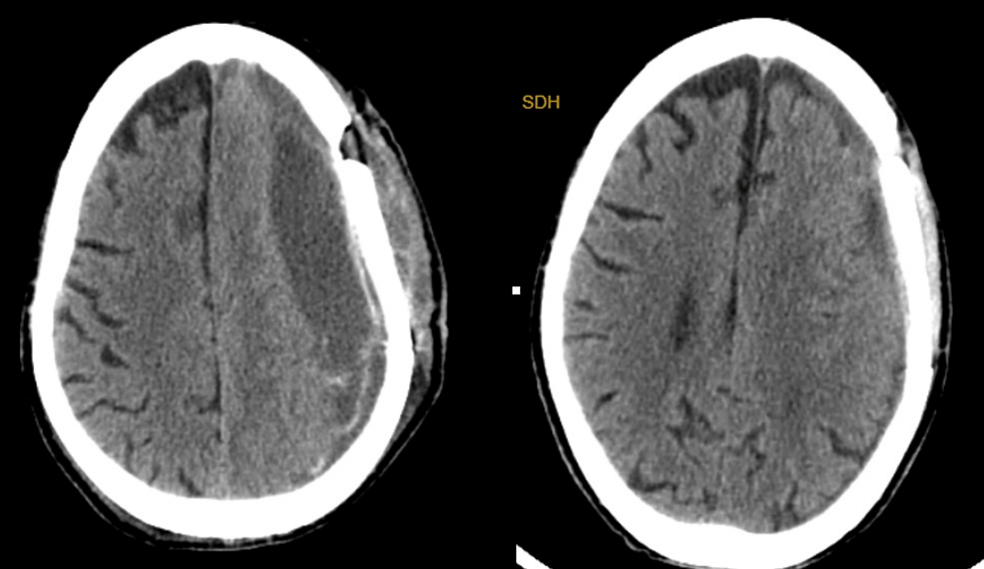
Recurrence after burr hole evacuation (left) treated by craniotomy (right)

A mixed type of CSDH was noted in 22 of the 32 recurrences, while the layering type was seen in seven cases. We had one case of hypodense type and two cases of isodense type with recurrences. Overall, the heterogenous subtypes (mixed and layering types) accounted for 29 of the 32 recurrences. There was a statistically significant correlation between recurrence and the heterogeneous subtypes with a p-value of 0.001. On multivariate analysis, heterogenous subtypes were a significant predictor of recurrence (OR: 8.88, 95% CI: 6.96-16.54, p = 0.01). The findings are summarized in Table 2. Figure 1 shows a case of recurrent CSDH treated by craniotomy.

**Table 2:** Radiological subtype and recurrence

	Recurrence	No recurrence	Total
Homogenous	3	90	93
Heterogenous	29	107	136
Total	32	197	229

Surgical risk factors

The mean duration between the first surgery and recurrence was noted to be 12.12 days. The initial surgical procedure was performed at the discretion of the operating surgeon. Among our recurrence group, 17 patients underwent evacuation with a single burr hole and 12 with two burr holes, and three patients underwent a mini-craniotomy as summarized in Table 3. Seven of the patients who experienced recurrence had a subdural drain, which was removed on postoperative day 2. There was no statistical correlation between the type of primary procedure and the recurrence rate (p-value = 0.4).

**Table 3:** Initial procedure and recurrence

	Recurrences	No recurrences	Total
Single burr hole	17	49	66
Two burr holes	12	108	120
Craniotomy	3	40	43
Total	32	197	229

Regarding those with recurrence, 24 patients underwent evacuation using two burr holes, with one placed in the frontal region and another in the parietal region. All of these patients had a subdural drain placed, which was removed on postoperative day 2. Four patients needed a third procedure after evacuation. The remaining 20 patients were discharged with good functional outcomes.

The remaining eight patients underwent a mini-craniotomy for evacuation. None of them needed another evacuation. However, a single patient expired during the postoperative period after a craniotomy.

The average length of postoperative hospital stays in the burr hole evacuation group after recurrence was 7.5 days, while it was 10.71 days for those who underwent craniotomy, excluding the one patient who expired after the second surgery.

Outcomes

We had four cases of refractory CSDH, all of whom underwent the second evacuation using burr holes. Three of them underwent evacuation via craniotomy, while the family of the fourth patient did not consent to the procedure. There was one case of wound infection that resolved with conservative care and antibiotics. Twenty-nine of the 31 patients were discharged and were able to resume their day-to-day activities.

## Discussion

While most cases of CSDH are amenable to surgery, recurrence remains an important complication. Several studies have attempted to identify the risk factors for recurrence leading to nearly 20 parameters considered to be relevant. This exemplifies the heterogeneity and complexity of the condition [12-14]. Studies have placed the chance of recurrence as high as 37% [15] with the average duration between the surgeries ranging from six days to 3.5 weeks [16,17]. In our study, the recurrence rate was 13.94% with the mean duration between recurrence being 12.12 days (six to 26 days).

While most studies have not found differences in recurrences between age, gender, comorbidities [7,18,19], use of antiplatelet drugs, or anticoagulant use, we found a correlation between gender and recurrence in line with the findings of Kim et al. [20]. This is probably due to the higher proportion of men in the study group. A trend toward recurrence in hypertensive patients was noted by Schwarz et al. in a study conducted in Germany [5]. A similar finding was noted in our study. Diabetes as a risk factor has yielded conflicting outcomes. Yamamoto et al. suggested a higher rate of recurrence in nondiabetics [16], while a study conducted by Mori et al. in a series of 500 patients with CSDH found a higher incidence of recurrence in diabetics [3]. In our study, diabetes did not increase the risk of recurrence.

Several radiological factors have been studied to see if they can serve as predictors of recurrence. Nomura et al. proposed a system based on imaging where the CSDH was grouped as either homogenous or heterogeneous. In our study, we found a statistically significant correlation between the Nomura subtype and recurrence [11]. They proposed that the higher density and the heterogeneity were due to cycles of hyperfibrinolysis and re-bleeding. Oishi et al. [6] have suggested that a higher rate of recurrence was noted with any degree of hyperdensity seen on CT scans. They hypothesize that this may indicate the presence of vulnerable capillaries within the neomembrane and have suggested withholding surgery till the hematoma becomes homogenous. However, they operated through a single burr hole, and clotted blood may not have been evacuated.

CSDH has a propensity to recur ipsilaterally or bilaterally with bilateral presentation considered a significant risk factor for recurrence [18,19]. We did find a similar finding in our study with a higher incidence of recurrence in bilateral CSDH. Another factor that was considered significant was the presence of midline shift, specifically a shift of more than 10 mm, which was considered a risk for recurrence [5,19]. Schawrz et al. noted an odds ratio of 2.69 with respect to a midline shift and recurrence. We noted a higher mean MLS in patients with recurrence. While some studies have found that the preoperative volume of CSDH was a predictor of recurrence [7], Huang et al. stated [21] that the preoperative volume has no bearing on the outcome, a finding shared by our study.

Weigel et al. [9] compared the various surgical means to address CSDH and found that craniotomies had the least recurrence (10.8%) at the cost of high morbidity (12.3%), while a single burr hole had the lowest morbidity (2.7%) at the cost of higher recurrence (12.1%). However, these studies were not randomized and are subject to bias. Jung et al. have reported no difference in recurrence rates between burr hole evacuation by one or two holes [19], while Smith et al. suggested a slight advantage with the use of two burr holes [22]. We found no difference in the rate of recurrence between the primary procedure done although there was a nonsignificant statistical trend toward less recurrence with craniotomies.

A significant factor enabling recurrence is the nonexpansion of the brain parenchyma. While the vascular outer membrane contributes to the bleeding and enlargement of the CSDH, the inner membrane, by adhering to the brain, prevents its expansion. One strategy has been to remove the avascular inner membrane gently so as to enable adequate expansion of the brain. This has also been thought to enable egress and reabsorption of the CSDH contents by the glymphatics and the dural lymphatics [23,24]. Since aggressive resection of the membrane can cause injury to the cortex and the veins, a fenestration has been recommended [25]. We did not notice any injury or adverse outcomes in those whose inner membranes were fenestrated.

Embolization of the middle meningeal artery has been described in the literature as a measure to address recurrences. Matsumoto et al. suggested the use of this technique for refractory CSDH in conjunction with surgery [26]. Their study presented a refractory rate of 2.4%. We had a refractory CSDH occurrence in 1.3% of our population. Cenic et al. [8] studied the preference of Canadian Neurosurgeons in treating recurrences and found a higher incidence of invasive procedures like craniotomy and subdural-peritoneal shunt. They found that evacuation using two burr holes and a craniotomy were the preferred methods in recurrence.

Thus, while multiple theories exist explaining the origin and propagation of CSDH, the most commonly accepted mechanisms include the presence of an active membrane that continues the cycle of fibrinolysis and bleeding with an inner membrane restricting the ability of the brain to expand, leading to the presence of a blood-filled space.

Limitations of the study

Being a single-center study involving multiple surgeons and the surgeon's preference on the type of surgery were the limitations of this study.

## Conclusions

Patient-related factors such as gender, bilateral presentation, and presence of hypertension and radiological factors such as the presence of heterogenous subtype and a significant midline shift are clues toward a higher chance of recurrence. Recurrence in CSDH can be adequately managed surgically with minimal morbidity and mortality. During craniotomy, the cautious removal of the outer and inner membranes may serve to prevent recurrence. However, even for recurrence, evacuation by means of two burr holes with a thorough lavage offers good results.

Other authors have identified several other risk factors demonstrating the heterogeneity of the condition, which warrants individualized decisions in the management of recurrent hematomas.
